# Deprescribing attitudes and predictors among older adults attending geriatric clinics in Kuwait

**DOI:** 10.1371/journal.pone.0311853

**Published:** 2024-12-19

**Authors:** Hesah Alshammari, Eman Al-Saeed, Zamzam Ahmed, Zoe Aslanpour

**Affiliations:** 1 Al-Sabah Hospital, Shuwaikh Medical Area, Ministry of Health, Kuwait City, Kuwait; 2 Department of Clinical and Pharmaceutical Sciences, School of Life and Medical Sciences, University of Hertfordshire, Hatfield, United Kingdom; 3 Courses and Programmes Director, The Organisation for Professionals in Regulatory Affairs (TOPRA), London, United Kingdom; Birjand University of Medical Sciences, ISLAMIC REPUBLIC OF IRAN

## Abstract

Deprescribing is defined as the reduction of medications to improve patient care. For effective deprescribing regular evaluation of medication adjustment regimens is required as it is documented to be an effective method to reduce polypharmacy and potentially inappropriate medications while improving patient well-being. Several factors, including patient-related aspects, influence the deprescribing process. Among these factors, patient willingness plays a pivotal role, making it essential to better understand their perspectives and attitudes towards medication use and deprescribing to successfully implement and maintain a deprescribing approach. We investigated the attitudes of older patients attending geriatric clinics in Kuwait toward deprescribing and identified predictors that influence their willingness to undergo this process. We enrolled patients aged ≥65 years who were attending geriatric clinics in primary care settings in Kuwait. These participants completed the revised Arabic version of the Patients’ Attitudes Towards Deprescribing (rPATD) questionnaire. The questionnaire was designed to assess the participants’ willingness to participate actively in medication decision-making and their inclination toward discontinuing certain medicines. Descriptive statistics was applied to gain insight into the characteristics of the participants and their responses to the rPATD questionnaire. Binary logistic regression identified predictors influencing the desire to deprescribe among participants. The study included 535 participants, out of which 388 were analyzed, with 233 (43.6%) being women. The majority, 77% (n = 412), were aged between 65 and 74 years. Out of the total, 205 patients (38.4%) had one to two medical conditions and were prescribed between one and five medications. The participants showed a high willingness to deprescribe, and this willingness was inversely associated with sex (p = 0.15), age (p = 0.15), and polypharmacy (p = 0.044). Many older patients visiting geriatric clinics in primary care settings in Kuwait were receptive to the concept of deprescribing medications, particularly if advised by their doctor. Nevertheless, it was observed that male patients, individuals on more than 5 medications, and older age groups showed lower willingness to deprescribe.

## Introduction

Potentially inappropriate medication (PIM) is a major health concern that significantly impacts the quality of life for individuals aged 65 and older. Although PIM can affect various age groups, it is most commonly observed in the older population. It has been documented that PIM is associated with adverse drug events, functional decline, morbidity, and mortality [1, 2]. A systematic review and a meta-analysis investigating PIM use and its association with adverse drug events, hospitalization, and mortality revealed that there is a statistically significant association between PIM use and adverse drug events and hospitalization (odds ratio [OR] = 1.44, 95% confidence interval [CI] = 1.33–1.56; OR = 1.27, 95% CI = 1.20–1.35) [3]. A considerable amount of literature globally has been published on the prevalence of PIM, and the results range from 34.7% to a staggering 80% [4–6]. Deprescribing is a systematic approach aimed at reducing or discontinuing medications that are no longer beneficial or may be harmful. It begins with an initial assessment to determine the appropriateness of current medications and involves regular follow-up to monitor and adjust treatment based on the patient’s evolving needs. This ongoing process requires continuous evaluation and adaptation to maintain effectiveness and address any emerging issues. Deprescribing is aided by several available tools, including Beer’s criteria, Screening Tool of Older Persons’ Prescriptions/ Screening Tool to Alert to Right Treatment (STOPP/START) and medication appropriateness index (MAI).

There is increasing evidence that deprescribing can reduce PIM and improve the health outcomes of older patients. A systematic literature review was carried out to investigate the impact and clinical outcomes of deprescribing as an intervention to reduce PIM in hospitalized older patients [7]. The review included only nine randomized controlled trials that were designed to reduce PIM in hospitals involving 2522 older patients. The review concluded that the deprescribing of PIM was statistically significant in seven studies. Nonetheless, a notable decrease in PIM was observed from admission to discharge in the intervention group.

Several factors hinder the optimization of older people’s medication and, more specifically, deprescribing inappropriate medicines. These factors can be grouped into two main categories: healthcare professionals and patients. Factors related to healthcare professionals include and are not limited to workload and time restrictions, resources, and knowledge and deprescribing skills [8–13]. The patient-related factors that influence deprescribing include patient expectations, fear of consequences, and attitudes toward deprescribing, and the influence of the caregiver [14–16].

One of the tools that were developed to explore the attitudes of older patients toward deprescribing is the Patient Attitudes Towards Deprescribing (PATD) questionnaire. It was initially developed in Australia in 2012 by Reeve et al. In 2016, a revised PATD (rPATD) was published for older adults and caregivers. Based on the review of studies that utilized the rPATD questionnaire to explore the attitudes of older patients [17–32], it can be concluded that deprescribing is a widely acceptable intervention for older adults ranging from 57% to 97%. The analysis indicates that several factors, such as medication burden and a strong relationship with healthcare providers, are associated with a higher willingness to deprescribe.

In general, deprescribing is widely accepted by older patients and their caregivers. However, the willingness to deprescribe among older patients could vary according to the health settings investigated [33]. To our knowledge, there are no known studies in the State of Kuwait or Gulf Council Cooperation (GCC) that have investigated older patients’ willingness to deprescribe. Therefore, this study aimed to explore the attitudes of older patients towards deprescribing and identify the predictors of willingness to deprescribe among older patients attending geriatric clinics in primary care centers (PCCs) in Kuwait.

## Methods

### Study location

Kuwait is a Middle Eastern country covering 17,820 km^2^, with an estimated population of around 4,947,468 people [34]. Its healthcare system is divided into both public and private sectors, with public healthcare structured into primary, secondary, and tertiary levels. Primary care is provided through approximately 97 PCCs distributed across the six governorates. In each governate, some of these centers include specialized geriatric clinics to cater to the needs of older adults. Secondary care is managed by six general hospitals, while tertiary care is handled by specialized hospitals and health centers. Medical services are free for Kuwaiti citizens, while non-citizens are required to pay nominal fees.

### Study design and sampling population

A multi-stage online and paper-based self-administered questionnaire targeted older patients (≥65 years) attending geriatric clinics in PCCs in Kuwait’s governorates. In order to reduce the selection bias, PCCs providing geriatric clinics were randomized using https://www.random.org/lists/ [35].

### Sampling strategy

Primary care centers offer walk-in appointments, therefore, patients who wish to visit the clinic must first obtain a visit number upon arrival. They are then required to wait until their number is called to see a doctor. As a result, a list of patient visits for the geriatric clinics was not available to randomly allocate the numbers to older patients. Therefore, convenient sampling was used to select participants. To ensure adequate representation of each governate, at least 64 participants were recruited from each PCCs across Kuwait.

The study included older adults aged 65 years and over who attended geriatric clinics. Participants who were attending other PCC clinics, such as those for chronic diseases or dental care, were excluded. No exclusions were made based on the number of prescribed medications or multimorbidity, allowing for a comprehensive assessment of the geriatric population attending these specialized clinics.

### Data collection tool

PATD tool was first developed in 2012 by Reeve et al. [36]. The questionnaire items were created after reviewing published qualitative studies exploring patients’ views on medications. In 2016, Emily Reeve et al. published a revised version of the PATD (rPATD) targeting older adults and caregivers. Nusair et al. translated rPATD into Arabic through forward-backward translation [37], guided by the professional society for health economics and outcomes research (ISPOR’s) principles of good practice for translation and cultural adaptation. The translated version was validated, and reliability was assessed using the Cronbach alpha coefficient and was considered acceptable with good internal consistency with an alpha ranging from 0.718 in the appropriateness domain to 0.850 in the concern domain. Furthermore, the test-retest of 32 participants gave a score of 0.718 to 0.972 for each item, which indicated good to excellent agreement between the groups. The questionnaire version developed by Nusair et al. was utilized in this study without any modifications.

The questionnaire was generated using Qualtrics, a University of Hertfordshire provided survey platform that supports Arabic for a simple writing of the questionnaire and identifying errors. A variety of distribution methods are available through Qualtrics; the link or barcode scan was used to access the questionnaire. Among the options implemented in the online questionnaire was the prevention of multiple responses from one device using an Internet Protocol (IP) address that is unique to each device.

### Statistical analysis

The questionnaire results were analyzed using descriptive statistics and statistical package for the social sciences (SPSS) version 27.0 for Windows. A 5-point Likert scale response to the statements of the Arabic rPATD questionnaire was dichotomized into those in agreement (agree and strongly agree) and those ambivalent or in disagreement (strongly disagree, disagree, and neither agree nor disagree). Three statements captured the willingness to deprescribe medications and were considered associated with primary outcomes related to attitude.

Appropriateness:

A2: I would like to try stopping one of my medications to see how I feel without it.
A3: I would like my doctor to reduce the dose of one or more of my medications.

Global:

G1: If my doctor said that it was possible, I would be willing to stop one or more of my regular medications.

After considering statements A2, A3 and G1, G1 statement has been chosen as the primary outcome measure because it reflects the overall patient’s willingness to stop taking their medications based on professional advice. This makes the G1 statement the most relevant and impactful measure of the primary outcome. To identify the willingness of the respondent to deprescribe, the responses to the primary outcome were dichotomized into two groups, agree and strongly agree, and neither agree nor disagree, disagree and strongly disagree, instead of the 5-point Likert scale. This was applied to the rest of the statements to perform forward conditional binary logistic regression.

### Sample size

The sample size was based on the assumption that 50% of the older population seeking medical treatment in PCC geriatric clinics. The 50% assumption, considered a conservative estimate, is a standard practice ensuring a sufficiently large sample size to detect significant effects, even when the true proportion is uncertain [38]. By choosing this figure, which represents the maximum variability, we aimed to ensure robust and reliable results [39]. This approach ensures that the sample size is adequate to generalize the findings and accounts for variability, making our study well-powered [40].

The sample size was calculated using EPI 7 software for the targeted population size of 150,592 older adults, ≥ 65 years, with a margin of error of 5%, and a confidence interval of 95% for six clusters. The sample size calculation software yielded a total of 384 participants with at least 64 participants in each governate in Kuwait that has a geriatric clinic in PCC. The decision to use 64 participants per governorate was deemed acceptable as it met the minimum sample size needed to ensure robust and representative results across the different governates in Kuwait. This distribution allowed for a balanced representation of each governorate while maintaining the overall study’s statistical power.

### Recruitment and questionnaire administration

The data collection process began on February 7^th^, 2021, and concluded on June 30th, 2021 during geriatric clinic operating hours, 8:00 AM to 1:00 PM. A multi-stage sampling was done to distribute the questionnaire. Initially, the questionnaire was distributed electronically. However, after the response rate was not high enough, the paper-based questionnaire was distributed to encourage more participation. This approach helped us to increase response rates and accommodate participants’ preferences or limitations regarding electronic access. The paper-based questionnaires were distributed in person by the researcher and clinic staff to ensure broader participation.

Upon arrival at the geriatric clinic, participants were briefed about the questionnaire. Participants were provided clear instructions on completing the questionnaire using their personal devices, with assistance from a doctor or researcher if necessary.

Each participant completed the questionnaire once, with data from a paper-based questionnaire entered into an electronic database between July 1st and July 8th. Consent was obtained in two ways, in the online questionnaire was indicated through a statement at the beginning of the questionnaire that agreeing to participate would be indicated through completing the questionnaire, and in the paper-based questionnaire participants provided written consent.

### Ethical approval

Ethical approval was obtained from the University of Hertfordshire (UH) (aLMS/PGR/UH/03951(2)) and from the Ministry of Health in Kuwait (2019/1154).

## Results

A total of 535 older patients responded to this questionnaire, 335 of which were recorded online and 200 were paper-based. A total of 116 and 31 online questionnaires and paper-based questionnaires, respectively, were excluded as they were incomplete, so the total number of questionnaires analyzed was 388. The online questionnaire had random locations of incomplete questions, but more than half (17 out of 31 incomplete surveys) of the paper-based questionnaires ended in the appropriateness domain. Part of the data set is in this manuscript’s S1 File.

### Characteristics of older patients

More than half of the questionnaire participants were women (n = 231, 59.5%) and the majority of the participants (n = 299, 77%) were aged 65–74 years and (85%) were Kuwaitis. Most of the participants could read and write (n = 151, 39%), had a bachelor’s degree (n = 114, 29.3%), or graduated from high school (n = 101, 26%), although only 5.7% (22 of 388) had a higher degree. Additionally, 52.8% (n = 205) of older adults had one or two medical conditions and were prescribed one to five medications. Table 1 shows the breakdown of the characteristics of the participants.

**Table 1:** Characteristics of the questionnaire participants stratified by sex.

		Sex				Total	
		Female		Male		Count	%
		Count	%	Count	%		
**Age**	65–74 years	190	48%	109	28%	299	(77%)
	75–84 years	39	10%	37	9.6%	76	(19.6%)
	≥85 years	2	0.5%	11	2.8%	13	(3.3%)
**Nationality**	Kuwaiti	198	51%	132	34%	330	(85%)
	Non-Kuwaiti	33	8.6%	25	6.4%	58	(15%)
**Education**	Able to read and write	107	27.6%	44	11.4%	151	(39%)
	High school	49	12.6%	52	13.4%	101	(26%)
	Bachelor’s degree	65	16.7%	49	12.6%	114	(29.3)
	Postgraduate degree	10	2.6%	12	3.1%	22	(5.7%)
**Number of medical conditions**	1–2	124	32%	81	20.8%	205	(52.8%)
	3–5	82	21.1%	60	15.5%	142	(36.6%)
	> 5	25	6.4%	16	4.2%	41	(10.6%)
**Number of prescribed medications**	1–2	46	11.9%	38	9.8%	84	(21.7%)
	3–5	86	22.1%	56	14.5%	142	(36.6%)
	6–10	67	17.3%	42	10.8%	109	(28.1%)
	11–15	27	7%	17	4.3%	44	(11.3%)
	16–20	3	0.77%	3	0.77%	6	(1.5%)
	>20	2	0.5%	1	0.3%	3	(0.8%)
**Have you been admitted to hospital within the last 12 months?**	Yes	82	21.2%	59	15.2%	141	(36.4%)
	No	149	38.4%	98	25.2%	247	(63.6%)

### Responses of older patients to the rPATD questionnaire

Most older adults (n = 253, 65.2%) reported taking a large number of medications and that taking medications every day was inconvenient (Table 2). However, most participants (n = 234, 60.3%) were reluctant to stop taking prescribed medications and several reported that they could miss future benefits (n = 199, 52.3%). Furthermore, most of the participants reported that they might feel stressed if changes were made to their medications (n = 240, 61.9%) and wanted to participate with their doctors in making decisions about altering their medications (n = 303, 78.1%).

**Table 2:** Patient responses to the Arabic rPATD questionnaire.

	Strongly agree		Agree		Neither agree nor disagree		Disagree		Strongly disagree	
	Count	%	Count	%	Count	%	Count	%	Count	%
***Burden***										
*I spend a lot of my money on medications*.	43	11.1%	104	26.8%	41	10.6%	128	33.0%	72	18.6%
*Taking my medicines every day is very inconvenient*	125	32.2%	128	33.0%	22	5.7%	81	20.9%	32	8.2%
*I feel like I am taking a large number of medications*.	113	29.1%	140	36.1%	25	6.4%	81	20.9%	29	7.5%
*I feel that my medications are a burden on me*.	107	27.6%	117	30.2%	44	11.3%	80	20.6%	40	10.3%
*Sometimes I think I take too many medicines*	114	29.4%	140	36.1%	34	8.8%	71	18.3%	29	7.5%
***Appropriateness***										
*I feel like I may be taking one or more medicines that I no longer need*.	55	14.2%	106	27.3%	89	22.9%	93	24.0%	45	11.6%
*I would like to try stopping one of my medications to see how I feel without it*.	72	18.6%	130	33.5%	75	19.3%	72	18.6%	39	10.1%
*I would like my doctor to reduce the dose of one or more of my medications*	94	24.2%	168	43.3%	62	16.0%	42	10.8%	22	5.7%
*I think one or more of my medications may not work*.	51	13.1%	119	30.7%	114	29.4%	70	18.0%	34	8.8%
*I believe one or more of my medications may be currently giving me side effects*	76	19.6%	140	36.1%	93	24.0%	59	15.2%	20	5.2%
**Concerns about stopping**										
*I would be reluctant to stop taking a medication that I had been taking for a long time*.	66	17.0%	168	43.3%	85	21.9%	53	13.7%	16	4.1%
*If one of my medicines was stopped, I would be worried about missing out on future benefits*	52	13.4%	147	37.9%	113	29.1%	57	14.7%	19	4.9%
*I get stressed whenever changes are made to my medications*.	78	20.1%	162	41.8%	55	14.2%	74	19.1%	19	4.9%
*If my doctor recommended stopping a medicine, I would feel that he/she was giving up on me*	24	6.2%	61	15.7%	111	28.6%	127	32.7%	65	16.8%
*I have had a bad experience when stopping a medicine before*	48	12.4%	105	27.1%	97	25.0%	100	25.8%	38	9.8%
***Involvement***										
*I have a good understanding of the reasons I was prescribed each of my medicines*	101	26.0%	188	48.5%	69	17.8%	25	6.4%	5	1.3%
*I know exactly what medications I am currently taking and / or I keep an up-to-date list of my medications*.	107	27.6%	201	51.8%	51	13.1%	26	6.7%	3	0.8%
*I like to know as much as possible about my medicines*	125	32.2%	209	53.9%	29	7.5%	23	5.9%	2	0.5%
*I like to be involved in making decisions about my medications with my doctors*.	122	31.4%	181	46.6%	48	12.4%	24	6.2%	13	3.4%
*I always ask my doctor*, *pharmacist*, *or other healthcare professional if there is something I don’t understand about my medications*.	140	36.1%	211	54.4%	22	5.7%	12	3.1%	3	0.8%
***Global***										
*If my doctor said it was possible*, *I would be willing to stop one or more of my regular medications*.	129	33.2%	185	47.7%	53	13.7%	13	3.4%	8	2.1%
*Overall*, *I am satisfied with my current medications*.	89	22.9%	180	46.4%	76	19.6%	29	7.5%	14	3.6%

Most older patients were satisfied with their medications (n = 269, 69.3%) and the statements reflecting their willingness to deprescribe were in agreement with most participants (Table 3). More than half of the participants wanted to stop one of the prescribed drugs and to see how they felt after discontinuation (n = 202, 52.1%). An observed increase in agreement was observed when the doctor participated in the decision to reduce at least one dose of their regular medicines (n = 262, 67.5%) or when the doctor suggested stopping one or more of their regular medications (n = 314, 80.9%).

**Table 3:** Responses of participants to statements reflecting their willingness to deprescribe.

		Age					
		65–74 years		75–84 years		≥85 years	
		Sex		Sex		Sex	
		Female	Male	Female	Male	Female	Male
		Count	Count	Count	Count	Count	Count
**I would like to try stopping one of my medicines to see how I feel without it**	**Agree**	109	54	19	14	2	4
	**Disagree**	81	55	20	23	0	7
**I would like my doctor to reduce the dose of one or more of my medicines**	**Agree**	140	63	31	21	2	5
	**Disagree**	50	46	8	16	0	6
**If my doctor said it was possible, I would be willing to stop one or more of my regular medications.**	**Agree**	166	87	31	24	1	5
	**Disagree**	24	22	8	13	1	6

### Older patient characteristics associated with willingness to deprescribe

Three patient characteristics were found to be statistically significant regarding the willingness to deprescribe if the doctors suggested deprescribing: male sex, older age, and being prescribed more than five medications were significantly associated with lack of willingness to deprescribe (Table 4). However, the level of education, governate, nationality and the number of medical conditions were not significantly associated with willingness to deprescribe, whereas the number of chronic medical conditions (P = 0.067) and history of being admitted to the hospital (P = 0.067) were close to significance.

**Table 4:** Association of patient characteristics and lack of willingness to deprescribe.

If my doctor said it was possible, I would be willing to stop one or more of my regular medications	P-value	OR	95% CI	
Sex (Male)	0.015	0.514	0.301	0.879
Age	0.015	0.557	0.347	0.892
Number of prescribed medications (>5)	0.044	0.768	0.595	0.993
Nationality	0.896			
Governate	0.896			
Education	0.402			
Number of medical conditions	0.080			
Have you been admitted to the hospital in the last 12 months?	0.067			

OR: Odds Ratio

CI: Confidence Interval.

## Discussion

This study was the first to explore attitudes towards deprescribing medications among older patients in the GCC and Kuwait. The study showed that most older adults were willing to have one or more of their regular medicines deprescribed if their treating doctor said it was possible, which is in line with several other studies conducted in different healthcare settings [18, 22, 23, 26]. However, other studies conducted in developed healthcare systems reported a higher willingness to deprescribe, reaching nearly 100% [30]. Context and individual factors may be responsible for variation in willingness to deprescribe [30].

Although most of the participants were willing to deprescribe one or more of the medications prescribed in this study, they were reluctant to discontinue a medication they had been taking for a long time. It is possible that this could preclude the deprescribing or the continuity of the intervention. The reluctance to discontinue prescribed long-term medications could be due to a variety of factors, for instance, the psychological attachment to a medication [41]. Furthermore, older patients tend to be reluctant to discontinue medications after taking them for a prolonged period of time due to their perceived severity of health conditions. Furthermore, from a doctor’s perspective, deprescribing antihypertensives to older adults could be stressful for patients who have experienced a stroke or a cardiac event, hence they refuse to participate in deprescribing [42]. This problem may be addressed by involving patients in health decision-making [43] but this might not be transferable to all long-term conditions and does not guarantee older patients will agree with the intervention. This is because there are many variables involved in this process, including attitudes, concerns of older patients, and the role of patient trust [43]. Improving older patient participation by providing adequate information about changes in the medication plan may be a factor associated with approving the decision to deprescribe [44].

In this study, the majority of the participants reported being stressed if changes were made to their medications. The stress related to changes in medication is a form of resistance to deprescribing [45, 46]. This stress is primarily rooted in the sensation of losing control over one’s medical condition due to alterations in medication [47]. Furthermore, stress associated with changes in medication can be attributed to a lack of trust in the healthcare provider and their decisions [45, 46]. It is important to understand the reasons underlying stress related to changes in medications in order to effectively deprescribe.

In this study, most of the participants reported their desire to participate in decision-making with their doctor. The literature documents that doctors perceive older patients as unwilling to be involved in decisions about their medication [48], contrary to what older patients reported in this study and the literature that they are willing to be involved in making health decisions with their doctor [24, 29, 32]. Active participation of older patients is the key to the successful implementation of a deprescribing.

Most of the participants were willing to deprescribe and had a generally positive attitude toward deprescribing, thus reassuring doctors about the general acceptability of deprescribing by older patients. However, several aspects must be considered when deprescribing, such as good communication and patient involvement in terms of explaining the rationale of deprescribing, the deprescribing plan, and preferably ways of contact in the event of any future concerns.

In this study, the high satisfaction of older patients with current medication accompanied the high willingness to deprescribe. This can be considered contradictory; however, referring to the Platonova et al. model of patient satisfaction, trust in doctors increases patient satisfaction with their current medication and any possible future intervention [49]. Good experience with the treating doctor and a longer relationship can be responsible for their satisfaction and accepting interventions [50].

Three characteristics of older patients were significantly associated with willingness to deprescribe, including age, sex, and polypharmacy. These characteristics were negatively associated with the willingness to deprescribe. First, increasing age was associated with unwillingness to deprescribe, which was consistent with Shrestha’s study conducted in Nepal which explored the attitudes of 385 older patients towards deprescribing [31]. This could be explained by attachment to medications or fear of stopping medications that have stabilized them well enough to continue living [41, 51]. This may also explain the link between polypharmacy and unwillingness to deprescribe. Lastly, women tended to be more accepting of the idea of deprescribing, while men were predicted to hinder the deprescribing process. This may be because men are prescribed more medications than women in Kuwait, which consequently can affect their decision to deprescribe [52]. Another possible explanation is that men tend to adhere more to prescribed medications than females [53], which is influenced by personality factors [54]. These significant characteristics tend to coexist, leading to higher resistance to deprescribing. Doctors and healthcare providers must be reminded of these characteristics to tailor and use different approaches to patient involvement and communication when targeting male or female patients, patients of advanced age, and older patients with polypharmacy.

Inconsistent findings have been reported in the international literature on the characteristics of older patients that predict the willingness to deprescribe. Several studies did not report any significant association between age, sex, number of medications, or willingness to deprescribe [19, 23, 25, 28, 29]. This inconsistent finding of predictors of unwillingness to deprescribe can be justified by the context and individual influence in willingness to deprescribe [33]. In Kuwait, cultural and religious beliefs may have an impact on attitudes toward deprescribing. The perception of health as divinely guided in a predominantly Muslim society can increase receptiveness to deprescribing when recommended by doctors [55, 56]. Additionally, beliefs about medication and fasting practices, such as those observed during Ramadan, influence attitudes toward medication adjustments. This can potentially lead to a high willingness to deprescribe [55, 56]. These cultural factors contribute to the differences observed in our study compared to findings from other geographical areas.

This is the first study to measure the attitudes of older patients towards deprescribing in Kuwait using a validated Arabic version of rPATD. This multicenter study involved a large number of older patients, and therefore a variety of statistical tests were possible. The study sample was divided according to governates to limit sampling bias. The recruitment of participants through diverse approaches, inclusive of distribution by geriatric clinic doctors and direct engagement by the researcher, facilitated the broad reaching of patients and the diminishment of sampling bias likelihood. To minimize response bias, participants were assured that their responses would remain confidential. The utilization of multiple data collection methods, encompassing both online and paper-based questionnaires, was instrumental in mitigating bias linked to a singular method. Additionally, stratification according to governate will enable exploring if it would influence the attitudes towards deprescribing. These study findings may help doctors understand the different patient characteristics that influence deprescribing.

The convenient sampling technique was a suitable method for recruiting older patients in the absence of older patient lists to enable randomization; however, it might produce limited generalizability of the findings. One of the limitations in this study is that the first stage of recruitment was via scanning barcodes and accessing the questionnaire, which could be difficult for older adults who are not familiar with such technology. Additionally, technical issues, such as unreliable internet connectivity, may hinder participation in the questionnaire. This study faces a limitation in that it lacks detailed reasoning behind participants’ willingness or unwillingness to deprescribe.

This study was conducted during the coronavirus disease of 2019, which may have influenced the distribution of the questionnaire and the data collection period. This study does not include caregiver attitudes, which are important to explore because they could influence the decision to deprescribing because the Arabic-translated version of rPATD did not include a caregiver section. The findings and interpretation of this study would greatly improve if caregiver attitudes toward deprescribing were investigated along with older patients.

This study suggests several implications for healthcare practice in Kuwait. Firstly, the Kuwaiti healthcare system needs to shift towards a patient-centred practice with a specific focus on patient engagement for successful deprescribing. Additionally, healthcare professionals need to improve their communication skills especially when discussing changes in medication regimens. Standardizing deprescribing protocols and providing comprehensive training to healthcare providers on the benefits and risks of deprescribing are essential steps to ensure a higher level of successful deprescribing. Lastly, it is essential to continuously research and evaluate deprescribing practices to improve care for older adults in Kuwait.

## Conclusions

Most older patients attending PCC settings in Kuwait governates were willing to deprescribe one or more of their medications if the doctor said it was possible. Several factors, such as male sex, older patients with polypharmacy and increasing age, were observed to be negatively associated with the willingness to deprescribe medications used for long term treatments.

## Supporting information

S1 File This file contains part of data collected in this study. (CSV)
